# Canine Parvovirus in Turkey: First Whole-Genome Sequences, Strain Distribution, and Prevalence

**DOI:** 10.3390/v15040957

**Published:** 2023-04-13

**Authors:** Mehmet Cevat Temizkan, Secil Sevinc Temizkan

**Affiliations:** 1 Department of Genetics, Faculty of Veterinary Medicine, Yozgat Bozok University, Yozgat 66700, Turkey; 2 Department of Virology, Faculty of Veterinary Medicine, Yozgat Bozok University, Yozgat 66700, Turkey; secil.s.temizkan@yobu.edu.tr

**Keywords:** canine parvovirus, genome, prevalence, sequencing, Turkey

## Abstract

Canine parvovirus (CPV) is a significant pathogenic virus with up to 100% morbidity and 91% mortality rates, especially in unvaccinated puppies. The emergence of new strains, interspecies transmission, and vaccine effectiveness can be enabled by just a few base changes in the CPV genome. Therefore, to cope with CPV disease, it is important to identify the viral agent and regularly monitor vaccine effectiveness against new strains. The present study has investigated CPV’s genetic profile in Turkey by collecting 80 samples from dogs in Turkey between 2020 and 2022. These samples and all sequences previously studied for CPV in Turkey were analyzed for whole-genome sequences, nationwide strain distribution over the two years, and the central Turkey prevalence rate. Next-generation sequencing was used for the genome study, Sanger sequencing for strain detection, and PCR for the prevalence analyses. The CPV-2 variants circulating in Turkey form their own cluster while being closely related to Egypt variants. Substantial amino acid changes were detected in antigenically important regions of the VP2 gene. Moreover, CPV-2b has become the most frequent genotype in this region, while the incidence of CPV-2c is predicted to increase gradually over the coming years. The prevalence of CPV in central Turkey was 86.27%. This study thus provides powerful insights to further our understanding of CPV’s genetic profile in Turkey and suggests that up-to-date vaccination efficacy studies are urgently needed.

## 1. Introduction

Canine parvovirus (CPV) 2 is a significant pathogenic virus with 100% morbidity at all ages, and 10% and 91% mortality in adult dogs and puppies, respectively [1,2]. Although clinical findings vary with age, the disease’s clinical signs generally include watery, odorous, and consistently bloody diarrhea, vomiting, weakness, depression, and anorexia. Particularly in newborn puppies, it causes nonsuppurative myocarditis that causes sudden death. Vaccination is thus crucial to prevent CPV-2 infection since unvaccinated puppies with CPV frequently die [3,4].

The International Committee on Taxonomy of Viruses (ICTV) classifies CPV-2 as a member of the *Parvoviridae* family, *Parvovirinae* subfamily, and *Protoparvovirus* genus. It is a small, non-enveloped virus with 5323 bases of single-stranded linear DNA. Reflecting CPV’s tendency to continuous evolution, three antigenic variants (CPV-2a, CPV-2b, and CPV-2c) have been reported [5,6,7]. The genome contains three major genes: the polyprotein non-structural protein-1 (NS1) and non-structural protein-2 (NS2) genes, the viral capsid protein-1 (VP1) gene, and the viral capsid protein-2 (VP2) gene. The VP2 region (especially the 426th amino acid) determines its antigenic properties, with mutations in this gene resulting in the emergence of antigenic variants [2,5,6,7]. Therefore, this gene directly determines vaccination efficacy, so amino acid changes detected in the VP2 gene have raised concerns about vaccination failures [8,9,10,11].

Molecular studies of Turkey’s dog population have detected CPV-2 antigenic variants [12,13,14,15,16,17,18,19,20,21,22,23]. These findings indicate that some antigenically important mutations are present in Turkey. Analyzed together, these studies can produce important results. Accordingly, the present study aims to determine the molecular and phylogenetic status of circulating strains of CPV in Turkey at the genome level, identify changes in CPV over the years, and measure its prevalence in the middle of Turkey.

## 2. Materials and Methods

### 2.1. Sample Collection

**Figure 1:**
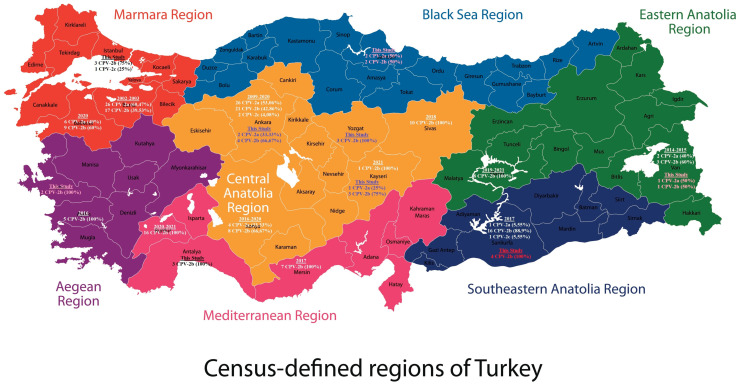
CPV distribution in Turkey.

For whole-genome (n = 5) and strain determination (n = 27) analysis, 32 feces samples were collected between 2020 and 2022 from the largest cities in seven regions in Turkey. These were Ankara (n = 6), Antalya (n = 3), Istanbul (n = 4), Izmir (n = 2), Samsun (n = 4), Sanliurfa (n = 4), Van (n = 2), Yozgat (n = 3), the central Turkish city where the study was conducted, and Kayseri (n = 4), a nearby city (Figure 1). Except for Yozgat, the samples were collected from animal shelters housing mixed-breed dogs that were unvaccinated or only vaccinated against rabies. The dogs were aged between 2 months and 4 years. For the whole-genome and strain determination analysis, feces were only collected from dogs showing CPV symptoms, such as bloody diarrhea, vomiting, and anorexia.

For the prevalence analysis, 51 feces samples were collected in 2022 from animal shelters in Yozgat, comprising 26 samples from puppies’ kennels, including 3 strain determination samples and 25 samples from the adult dogs’ kennels. These 51 samples were used for PCR assays and classified as CPV-positive or -negative except for 3 strain determination samples. The sampled dogs were unvaccinated or only vaccinated against rabies, mixed-breed, and aged between 2 to 6 months and 1 to 5 years in puppies and adult dogs, respectively. While 24 of the puppies had diarrhea, none of the adult dogs had symptoms. The vitality of the dogs was checked by observation one month after sampling. No treatment was provided by the shelter during this period due to economic constraints.

All samples were collected in sterile stool containers before being transported on dry ice to the Genetics Laboratory, Faculty of Veterinary Medicine, Yozgat Bozok University, and stored at −20 °C until DNA isolation.

### 2.2. DNA Isolation

For the whole-genome and strain determination analysis, DNA isolation was performed using the QIAamp Cador Pathogen Mini Kit (Qiagen, Hilden, Germany). For the prevalence analysis, DNA isolation was performed using the QIAamp DNA Stool Mini Kit (Qiagen, Hilden, Germany) according to the manufacturer’s protocols. All DNA isolations were performed at the Genetics Laboratory, Faculty of Veterinary Medicine, Yozgat Bozok University. The isolates were then stored at −20°C until the PCR analysis.

### 2.3. PCR Analysis

The samples were transported in dry ice to the Molecular Biology and Genetics Laboratory for PCR analysis, Faculty of Veterinary Medicine, Konya Selcuk University. This was performed with CPV-specific primers designed for this study to amplify the 426th amino acid on the VP2 gene. For the PCR analysis, the following F and R primers were used, respectively: AAACTACCACAACAGGAGAAACAC and TGGTGCATTTACATGAAGTCTTGG. These were expected to amplify a 302 bp region in the CPV genome. PCR was performed in a T Professional Thermal Cycler (Biometra, Göttingen, Germany) using DreamTaq Polymerase (Thermo Scientific, Waltham, MA, USA, #EP0702). The reaction mix was prepared as follows per 2.5 μL DNA sample: 2.5 μL 10X DreamTaq Buffer, 0.5 μL 10 mM dNTP and 0.5 μL for each primer, 0.25 μL (5U/μL) Dream Taq DNA Polymerase, and 18.25 μL nuclease-free water. PCR was performed according to the following protocol: 95 °C for 3 min, 35 cycles of 95°C for 30 s, 61 °C for 30 s, 72 °C for 1 min, and 72 °C 1 min for the final extension.

Agarose gel (1%) was prepared to visualize the amplified PCR products, which were inspected in a Gel Logic 100 imaging device (Kodak, New York, NY, USA).

### 2.4. Sanger Sequencing

For the strain determination analysis, Sanger sequencing of the PCR products (27 samples from 9 cities) was performed by a commercial company (BM Labosis, BM Lab. Schist. Ltd. Sti. Ankara, Turkey). The samples were transported on dry ice to the company headquarters in Ankara. The obtained sequences were identified using BLAST, provided on the NCBI web page. After alignment based on the VP2 gene, the sequences were translated into amino acids with MEGA X and AliView software using the CPV reference sequence (NC001539) from the GenBank database. The obtained sequences were submitted to the GenBank database (Supplement S1).

### 2.5. Next-Generation Sequencing

For the whole-genome analysis, five samples were sequenced from Ankara, Izmir (two samples), Samsun, and Sanliurfa. The samples were transported in dry ice to the CUTAM Laboratory, Sivas Cumhuriyet University, for next-generation sequencing. Sequencing was performed in an Illumina MiSeq device using a Nextera XT DNA Library Preparation Kit to create the DNA library. The CPV genome data were extracted with Bowtie 2 software [24] and aligned with the reference genome NC001539 using MAFFT multiple sequence alignment software [25]. An average of 12,000 of 500,000 reads were assembled to reference. The obtained genomes were submitted to the GenBank database (Supplement S1).

### 2.6. Sequence Selection and Phylogenetic Analysis

For the whole-genome phylogenetic analysis, nearby countries were selected first, followed by more distant countries as the basis. Two genomes that had been previously reported in Turkey were also added [12]; 43 sequences were taken from GenBank data (Supplement S2) and previous studies [26,27,28,29,30,31,32,33,34,35,36,37,38,39,40,41,42].

All CPV sequences obtained from the present study, 174 GenBank sequences in Turkey until this date (Supplement S1), and previous studies [12,13,14,15,16,17,18,19,20,21,22,23] are listed according to year and sampling location (Figure 1; Supplement S1). The phylogenetic analyses of VP2 in Turkey include only 31 whole VP2 sequences, including the present study’s samples (whole VP2 gene from 5 whole genomes) (Supplement S1). Partial VP2 sequences were only used to analyze strain variation over the years in Turkey. They were excluded from the phylogenetic analysis as they contained different and small amplified regions. To perform strain determination and phylogenetic analyses with CPV strains seen in cats and wolves in Turkey, a second analysis was performed using GenBank (OM805994) CPV cat and wolf isolates and shortened whole VP2 sequences (60-1,630 VP2 gene nucleotides), as previously described [43,44,45]. Only seven samples were found to be of sufficient length for the phylogenetic analysis of the cat and wolf strains [43,44].

The obtained genomes and VP2 sequences were aligned and phylogenetically analyzed with MEGA X [46] and AliView software. These were used together based on the reference sequence NC001539. The phylogenetic trees were constructed using the neighbor-joining method with 1000 bootstrap replicates and the p-distance parameter model in MEGA X [40].

## 3. Results

**Figure 2:**
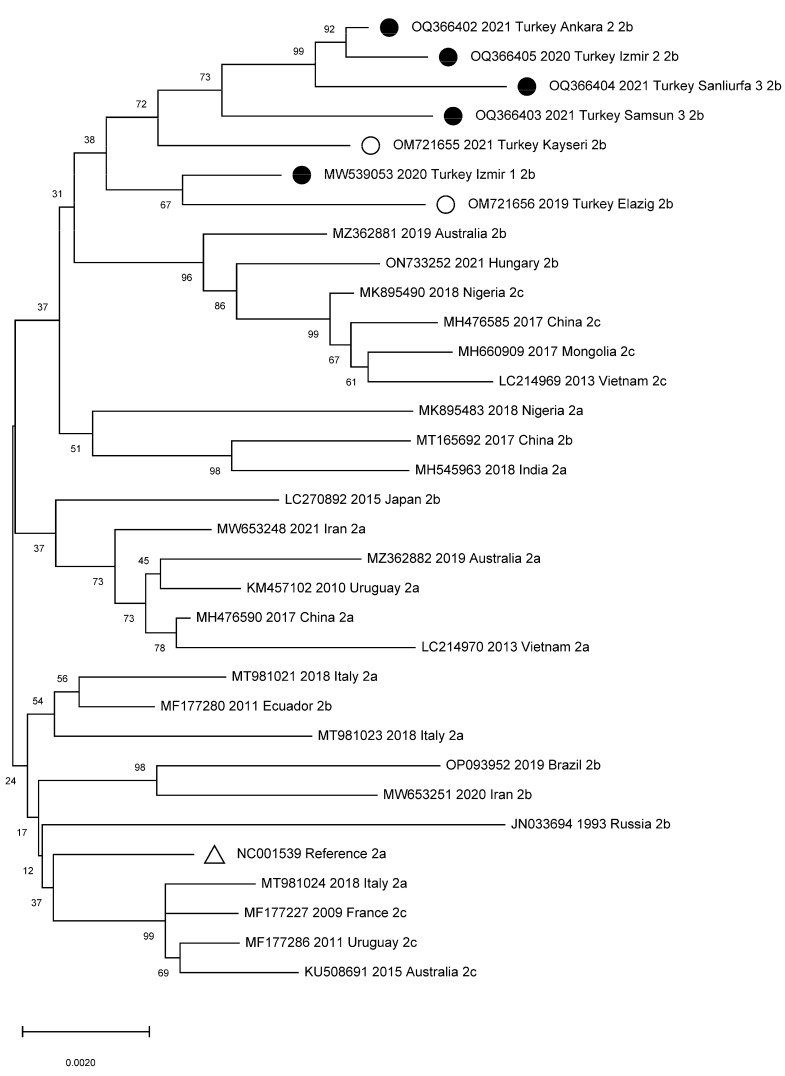
Phylogenetic tree based on CPV-2 genomic sequences worldwide. ●: Samples collected in this study from Turkey. ○: Samples collected in the previous study from Turkey. ∆: CPV reference genome.

**Figure 3:**
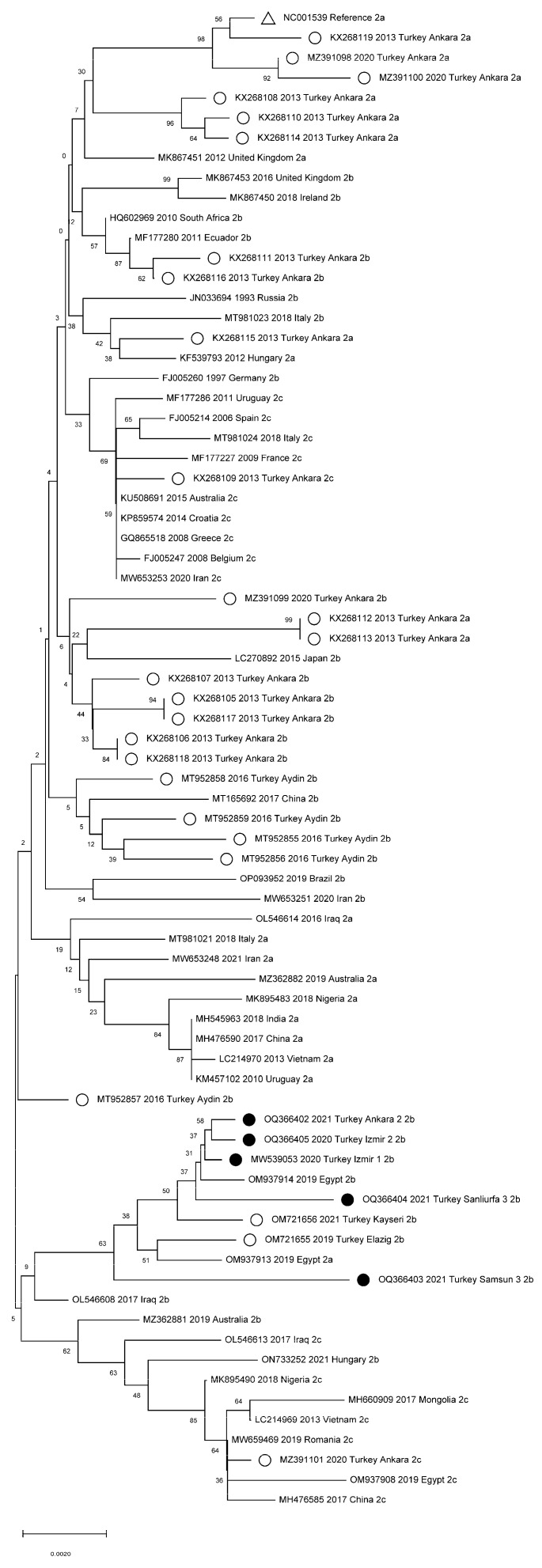
Phylogenetic tree based on the VP2 gene worldwide. ●: Samples collected in this study from Turkey. ○: Samples collected in the previous study from Turkey. ∆: CPV reference genome.

Figure 2 shows the results of the comparative phylogenetic analysis of previous CPV whole-genome sequence studies worldwide and in Turkey. Phylogenetic trees were constructed for the VP2 (Figure 3), VP1, and polyprotein NS1/NS2 genes (Supplement S3) and for the cat, wolf, and dog VP2 sequences circulating in Turkey (Supplement S4).

A comparison of the sequencing results with the reference genome (NC001539) indicated the presence of 141 nucleotide variations (Supplement S5). Together with previously published studies of CPV in Turkey, 28, 23, and 14 amino acid differences were found in the VP2 (Table 1), VP1 (Table 2), and NS1/NS2 (Table 3) genes, respectively.

The whole-genome and VP2 sequences submitted to GenBank in Turkey so far are presented in Supplement S1, while the annual strain changes of all sequences according to previous literature and GenBank are presented in Table 4, respectively. According to all sequences obtained in Turkey so far, 25.86% (45/174) of the samples were CPV-2a, 71.84% (125/174) were CPV-2b, and 2.3% (4/174) were CPV-2c (Supplement S1). The first strain determination studies in Turkey, conducted between 2002–2003, found that 60.47% (26/43) of the samples were CPV-2a and 39.53% (17/43) were CPV-2b [22,23]. Lattermost, strain determination studies conducted in Turkey between 2020 and 2022 found that 17.72% (14/79) of the samples were CPV-2a, 79.75% (63/79) were CPV-2b, and 2.53% (2/79) were CPV-2c (Supplement S1). In the present study, 18.75% (6/32) of the samples were CPV-2a, 78.13% (25/32) were CPV-2b, and 3.12% (1/32) were CPV-2c (Figure 1). In CPV strains isolated from cats between 2009 and 2010, 88.89% (8/9) of the samples were CPV-2a and 11.11% (1/9) were CPV-2c. However, all samples (6/6) identified from the previous literature [43] and GenBank (OM805994) between 2017 and 2021 were CPV-2b.

In the present study, CPV prevalence in Yozgat province was 86.27% (44/51) overall and 100% (26/26) and 72% (18/25) in puppies and adult dogs, respectively. While 96.15% (25/26) of puppies had died within one month after sampling, there were no deaths among the adult dogs.

## 4. Discussion

The whole-genome phylogenetic analysis results show that current variants circulating in Turkey form their own cluster (Figure 2). This may be because of the diversification of the virus circulation within the country due to a large number of stray dogs without CPV vaccination. The recently characterized VP2 gene sequences in Turkey are closely related to those detected in Egypt [34]. However, since no whole-genome sequence study has been conducted in Egypt, a genome-level comparison could not be made. Our results also suggest that the CPV-2b variations currently seen in both Turkey and Egypt had spread to these two countries from Iraq (Figure 3). While the VP1 gene results were similar to the whole-genome results, in two samples, the results for the NS1/NS2 genes differed from the whole-genome results. This indicates that the NS1/NS2 genes are better preserved (Supplement S3), in line with previous reports [8]. The phylogenetic tree for cats and wolves shows that the CPV variants circulating among cats in Turkey form a separate branch. However, the detected strains have completely changed over time from CPV-2a and CPV-2c to CPV-2b [43,45]. CPV strains found in wolves were found to be closely related to strains detected in dogs recently (Supplement S4) [44].

The amino acid differentiation variant patterns in our study are compatible with previous studies in both Turkey and other countries. The analysis shows that mutations and polymorphisms in this study’s whole-genome sequences caused changes in the critical residues of the viral proteins (Table 1). Previous studies have revealed that changes in the 5th, 87th, 101st, 267th, 297th, 300th, 305th, 324th, 370th, 426th, and 440th amino acid residues of the VP2 protein are important for antigenicity [8,9,10,11]. A5G and Q370R mutations are suspected to be the host range and interaction between the host DNA and VP2 protein [8]. Two samples from Turkey had A5G (MZ391101, OM721656), while one had both A5G and Q370R mutations (MZ391101). Amino acid changes in residues F267Y, Y324I, and T440A may indicate a new subvariant, while the combination of these three changes may explain vaccination failures. However, the role of the 267th amino acid is still unclear, although it is an unexposed residue [11,47]. The 324th and 440th amino acids are located next to the 426th and 427th spike residues, respectively [47]. The F267Y-Y324I-T440A mutation combination has also been detected in dog populations in Ankara, Elazig, Izmir, and Kayseri (Table 1). According to Mittal et al. [10], changes in the S297A, Y324I, and T440A residues are important for host adaptation, host range, and antigenic importance, respectively, and may reduce vaccine efficacy. This combination has also been detected in Turkey dog populations in Ankara, Elazig, Izmir, Kayseri, and Samsun. Several other unique VP2 gene mutations have also been detected in Turkey (Table 1). Our results for the VP1 and polyprotein NS1/NS2 genes are similar to previous studies worldwide [26,27,28,29,30,31,32,33,34,35,36,37,38,39,40,41,42]. In the present study, several unique mutations were detected in the VP1 (2AP, Q135H, F196C, T408I, Y467V) and NS1/NS2 (I248T, V540I, S576L, C579Y, L582S, E583K, Q595E) genes in Turkey (Table 2 and Table 3).

Our results clearly show that CPV-2b has become the most common variant in Turkey (Table 4), followed by CPV-2a and CPV-2c. All variants detected to date in the Mediterranean and Aegean regions of Turkey are CPV-2b (n = 33). Moreover, no CPV-2c variants have been observed in the Black Sea and Eastern Anatolian regions (n = 19) (Figure 1). The CPV-2c variant detected in Istanbul in our study is only the fourth such variant found in dogs in Turkey. Considering CPV-2c’s rapid global spread [8,34] and Turkey’s touristic potential, CPV-2c prevalence is predicted to increase gradually in Turkey in the coming years.

Our alignment results for CPV-2c were unexpected because an earlier study of Turkey’s dog population reported that the first CPV-2c was sequenced in 2017. Thus, it was thought that the sequence accession number MG780282 was the first CPV-2c genotype seen in Turkey, and it was accepted as indicating CPV-2c’s first appearance in this dog population [20]. Unfortunately, we found that this information was incorrect as our alignment results revealed that sequence accession number KX268109, which includes the CPV-2c genotype, was collected in 2013, sequenced in 2015 [16], and submitted to the NCBI in 2016 but not published. In short, the first appearance of the CPV-2c genotype in Turkey’s dog population should be updated to 2013. Abayli et al. [12] did not include that information on CPV-2c and provided the first complete genomic analysis of CPV-2 isolates (OM721655 and OM721656). However, this information was also incorrect as our sequence MW539053 revealed that the sample was collected in 2020 and sequenced, submitted, and presented in 2021 [34]. It was made available to the NCBI in 2022, six months before the OM721655 and OM721656 submissions to GenBank. Lastly, Hasircioglu and Aslim [48] reported CPV-2a, CPV-2b, and CPV-2c genotypes together in 26 of 30 CPV-positive animals. Since our sequencing results show that this cannot be possible, we omitted their study from our analysis results.

The prevalence results show that CPV disease has become quite severe in central Turkey as the mortality rate in the sampled CPV-infected puppies was 96.15%, which is similar to previous reports [1,2]. These results show that CPV remains an important cause of puppy mortality in Turkey, so CPV vaccination programs should be urgently started in animal shelters to control the disease.

To sum up, current CPV-2 variants circulating in Turkey have their own whole-genome phylogenetic cluster and are closely related to Egypt variants in terms of the VP2 gene. We also detected amino acid changes in antigenically important regions of the VP2 gene. CPV-2b has clearly become the most common genotype in Turkey, although CPV-2c prevalence is predicted to increase gradually in Turkey in the coming years. We also recommend revising the determination date for the first CPV-2c genotype seen in Turkey from 2017 to 2013. Our prevalence results show that CPV remains highly dangerous for puppies in central Turkey, with a mortality rate of up to 96.15%. We, therefore, suggest that future work should urgently change focus towards evaluating CPV vaccination efficacy and implementing CPV vaccination programs in animal shelters.

## Figures and Tables

**Table 1:** VP2 gene’s amino acid variations.

**VP2 Gene’s Amino Acid Variations**																
Amino Acid No.	5	13	40	44	52	53	55	68	87	101	156	256	265	267	271	297
NC001539_Ref	A	P	I	T	K	F	E	L	M	I	S	R	T	F	K	S
OQ366402_Ankara_2_2b	A	P	I	T	K	F	E	L	L	T	S	R	T	Y	K	A
MW539053_İzmir_1_2b	A	P	I	T	K	F	E	L	L	T	S	R	T	Y	K	A
OQ366405_İzmir_2_2b	A	P	I	T	K	F	E	L	L	T	S	R	T	Y	K	A
OQ366403_Samsun_3_2b	A	P	M	S	K	C	E	F	L	T	S	R	T	F	K	A
OQ366404_Sanliurfa_3_2b	A	P	I	T	K	F	E	L	L	T	S	R	I	Y	K	A
OM721656_Kayseri_2b	G	S	I	T	K	F	E	L	L	T	S	R	T	Y	K	A
OM721655_Elazig_2b	A	P	I	T	T	F	Q	L	L	T	S	R	T	Y	K	A
KX268105_Ankara_2b	A	P	I	T	K	F	E	L	L	T	S	R	T	F	K	A
KX268106_Ankara_2b	A	P	I	T	K	F	E	L	L	T	S	R	T	F	K	A
KX268107_Ankara_2b	A	P	I	T	K	F	E	L	L	T	S	R	T	F	K	A
KX268108_Ankara_2a	A	P	I	T	K	F	E	L	L	T	S	R	T	F	K	A
KX268109_Ankara_2c	A	P	I	T	K	F	E	L	L	T	S	R	T	F	K	A
KX268110_Ankara_2a	A	P	I	T	K	F	E	L	L	T	S	R	T	F	K	A
KX268111_Ankara_2b	A	P	I	T	K	F	E	L	L	T	S	R	T	F	K	A
KX268112_Ankara_2a	A	P	I	T	K	F	E	L	L	T	S	R	T	F	K	A
KX268113_Ankara_2a	A	P	I	T	K	F	E	L	L	T	S	R	T	F	K	A
KX268114_Ankara_2a	A	P	I	T	K	F	E	L	L	I	S	R	T	F	K	A
KX268115_Ankara_2a	A	P	I	T	K	F	E	L	L	T	S	R	T	F	K	A
KX268116_Ankara_2b	A	P	I	T	K	F	E	L	L	T	S	R	T	F	K	A
KX268117_Ankara_2b	A	P	I	T	K	F	E	L	L	T	S	R	T	F	K	A
KX268118_Ankara_2b	A	P	I	T	K	F	E	L	L	T	S	R	T	F	K	A
KX268119_Ankara_2a	A	P	I	A	K	F	E	L	M	I	S	R	T	F	K	S
MT952855_Aydin_2b	A	P	I	T	K	F	E	L	L	T	S	K	T	Y	K	A
MT952856_Aydin_2b	A	P	I	T	K	F	E	L	M	T	S	R	T	Y	K	A
MT952857_Aydin_2b	A	P	I	T	K	F	E	L	L	T	S	R	T	Y	K	T
MT952858_Aydin_2b	A	P	I	T	K	F	E	L	L	T	S	R	T	Y	K	A
MT952859_Aydin_2b	A	P	I	T	K	F	E	L	M	T	S	R	T	Y	K	A
MZ391098_Ankara_2a	A	P	I	A	K	F	E	L	M	I	S	R	T	F	K	S
MZ391099_Ankara_2b	A	P	I	T	K	F	E	L	M	T	F	R	T	F	K	A
MZ391100_Ankara_2a	A	P	I	A	K	F	E	L	M	I	S	R	T	F	R	S
MZ391101_Ankara_2c	G	P	I	T	K	F	E	L	L	T	S	R	T	Y	K	A
**VP2 Gene’s Amino Acid Variations**																
Amino Acid No.	300	301	305	316	324	367	370	375	426	440	573	580	References			
NC001539_Ref	A	T	D	V	Y	Y	Q	D	N	T	Y	P				
OQ366402_Ankara_2_2b	G	T	Y	V	I	D	Q	D	D	A	Y	P	This Study			
MW539053_İzmir_1_2b	G	T	Y	V	I	D	Q	D	D	A	Y	P				
OQ366405_İzmir_2_2b	G	T	Y	V	I	D	Q	D	D	A	Y	P				
OQ366403_Samsun_3_2b	G	T	Y	V	I	D	Q	D	D	A	Y	P				
OQ366404_Sanliurfa_3_2b	G	T	Y	V	V	D	Q	D	D	A	Y	P				
OM721655_Kayseri_2b	G	T	Y	V	I	D	Q	D	D	A	Y	P	[12]			
OM721656_Elazig_2b	G	T	Y	V	I	D	Q	D	D	A	Y	P				
KX268105_Ankara_2b	G	T	Y	V	Y	D	Q	D	D	T	Y	P	[16]			
KX268106_Ankara_2b	G	T	Y	V	Y	D	Q	D	D	T	Y	P				
KX268107_Ankara_2b	G	T	Y	V	Y	D	Q	D	D	T	Y	P				
KX268108_Ankara_2a	G	T	Y	V	Y	D	Q	D	N	A	Y	P				
KX268109_Ankara_2c	G	T	Y	V	Y	D	Q	D	E	T	Y	P				
KX268110_Ankara_2a	G	T	Y	V	Y	D	Q	D	N	A	Y	P				
KX268111_Ankara_2b	G	T	Y	V	Y	D	Q	D	D	T	Y	P				
KX268112_Ankara_2a	G	T	Y	V	Y	D	Q	D	N	T	Y	P				
KX268113_Ankara_2a	G	T	Y	V	Y	D	Q	D	N	T	Y	P				
KX268114_Ankara_2a	G	T	Y	V	Y	D	Q	D	N	A	Y	P				
KX268115_Ankara_2a	G	T	Y	V	Y	D	Q	D	N	T	Y	P				
KX268116_Ankara_2b	G	T	Y	V	Y	D	Q	D	D	T	Y	P				
KX268117_Ankara_2b	G	T	Y	V	Y	D	Q	D	D	T	Y	P				
KX268118_Ankara_2b	G	T	Y	V	Y	D	Q	D	D	T	Y	P				
KX268119_Ankara_2a	A	T	D	V	Y	D	Q	E	N	T	F	P				
MT952855_Aydin_2b	G	T	Y	V	Y	D	Q	N	D	A	Y	S	GenBank (Saltik and Koc, 2020)			
MT952856_Aydin_2b	G	T	Y	V	Y	D	Q	D	D	T	Y	P				
MT952857_Aydin_2b	G	T	Y	V	I	D	Q	D	D	T	Y	P				
MT952858_Aydin_2b	G	T	Y	V	Y	D	Q	D	D	T	Y	P				
MT952859_Aydin_2b	G	T	Y	V	N	D	Q	D	D	T	Y	P				
MZ391098_Ankara_2a	A	T	D	N/A	Y	D	Q	N	N	T	Y	P	GenBank (Kizilkoca and Tan, 2021)			
MZ391099_Ankara_2b	G	T	Y	V	Y	D	Q	D	D	T	Y	P				
MZ391100_Ankara_2a	D	I	D	L	Y	D	Q	N	N	T	Y	P				
MZ391101_Ankara_2c	G	T	Y	V	I	D	R	D	E	T	Y	P				

The gray-shaded amino acids are different from the reference.

**Table 2:** VP1 gene’s amino acid variations.

**VP1 Gene’s Amino Acid Variations**																
Amino Acid No.	2	114	116	131	135	148	156	183	187	195	196	211	230	244	408	410
NC001539_Ref	A	R	K	A	Q	A	P	I	T	K	F	L	M	I	T	F
OQ366402_Ankara_2_2b	P	R	K	A	Q	A	P	I	T	K	F	L	L	T	T	Y
MW539053_İzmir_1_2b	A	R	R	A	Q	A	P	I	T	K	F	L	L	T	T	Y
OQ366405_İzmir_2_2b	P	R	K	A	H	A	P	I	T	K	F	L	L	T	T	Y
OQ366403_Samsun_3_2b	P	R	K	A	Q	A	P	M	S	K	C	F	L	T	T	F
OQ366404_Sanliurfa_3_2b	P	R	R	A	Q	A	P	I	T	K	F	L	L	T	I	Y
OM721655_Kayseri_2b	A	R	K	A	Q	A	P	I	T	T	F	L	L	T	T	Y
OM721656_Elazig_2b	A	K	K	T	Q	G	S	I	T	K	F	L	L	T	T	Y
**VP1 Gene’s Amino Acid Variations**																
Amino Acid No.	440	443	448	467	510	569	583	References								
NC001539_Ref	S	A	D	Y	Y	N	T									
OQ366402_Ankara_2_2b	A	G	Y	I	D	D	A	This study								
MW539053_İzmir_1_2b	A	G	Y	I	D	D	A									
OQ366405_İzmir_2_2b	A	G	Y	I	D	D	A									
OQ366403_Samsun_3_2b	A	G	Y	I	D	D	A									
OQ366404_Sanliurfa_3_2b	A	G	Y	V	D	D	A									
OM721655_Kayseri_2b	A	G	Y	I	D	D	A	[12]								
OM721656_Elazig_2b	A	G	Y	I	D	D	A									

The gray-shaded amino acids are different from the reference.

**Table 3:** NS1/NS2 genes’ amino acid variations.

NS1/NS2 Genes’ Amino Acid Variations															
Amino Acid No.	248	255	307	309	375	540	544	560	576	579	582	583	595	597	References
NC001539_Ref	I	E	Q	D	W	V	Y	E	S	C	L	E	Q	L	
OQ366402_Ankara_2_2b	I	E	Q	D	W	V	Y	D	S	C	S	K	Q	L	This study
MW539053_İzmir_1_2b	I	E	Q	D	W	V	F	E	S	C	L	E	Q	P	
OQ366405_İzmir_2_2b	I	E	Q	D	W	I	Y	D	S	C	S	K	Q	L	
OQ366403_Samsun_3_2b	T	E	Q	D	W	V	Y	D	L	C	L	E	E	L	
OQ366404_Sanliurfa_3_2b	I	E	Q	D	W	V	Y	D	S	Y	S	K	Q	L	
OM721655_Kayseri_2b	I	E	Q	E	G	V	Y	D	S	C	L	E	Q	L	[12]
OM721656_Elazig_2b	I	G	T	D	W	V	F	E	S	C	L	E	Q	P	

The gray-shaded amino acids are different from the reference.

**Table 4:** Change of CPV strains in Turkey 2002–2022.

Sampling Year	Sample Number	CPV-2a	CPV-2b	CPV-2c	Location	References
2002–2003	43	60.47%	39.53%	0%	Bursa (Not sequenced)	[22,23]
2009–2010	25	68%	32%	0%	Ankara	[21]
2013–2015	20	45%	50%	5%	Ankara, Van	[16,19]; GenBank (Karapinar, 2015)
2016–2017	38	13.16%	84.21%	2.63%	Aydin, Konya, Mersin, Sanliurfa	[14,15,20]; GenBank (Saltik and Koc)
2018–2019	19	0%	100%	0%	Ankara, Elazig, Sivas	[12,13,18]
2020	44	18.18%	79.55%	2.72%	Ankara, Balıkesir, Burdur, Elazig, Izmir, Konya	[12,17] GenBank (Kizilkoca and Tan, 2021); GenBank (Karapinar and Timurkan, 2021); GenBank (Dik et al., 2021); this study
2021	21	19.05%	80.95%	0%	Ankara, Burdur, Elazig, Kayseri, Samsun, Sanliurfa, Yozgat	[12]; GenBank (Hasircioglu, 2022); this study
2022	14	14.29%	78.57%	7.14%	Antalya, Istanbul, Kayseri, Van, Yozgat	This study
Total	224	31.70%	66.51%	1.79%	All	

## Data Availability

The obtained data of sequences from this study were deposited in the GenBank database with the Accession Numbers: MW539053, OQ355596-622, OQ366402-05 (Supplement S2).

## Supplementary Materials

The following supporting information can be downloaded at: https://www.mdpi.com/article/10.3390/v15040957/s1, Supplement S1: Entire CPV Genbank accession numbers from dogs in Turkey. Supplement S2: Worldwide CPV genomes and VP2 gene sequences from dogs. Supplement S3: Phylogenetic tree based on NS1/NS2 and V1 genes worldwide, (A) VP1 gene (B) polyprotein NS1/NS2 genes, ●: Samples collected in this study from Turkey, ○: Samples collected in the previous study from Turkey, ∆: CPV reference genome. Supplement S4: Phylogenetic tree based on cat, wolf, and dog sequences in Turkey, ●: Samples collected in this study from Turkey, ■: Samples collected from cats in the previous study from Turkey, ▲: Samples collected from wolves in the previous study from Turkey, ○: CPV reference genome. Supplement S5: CPV whole-genome nucleotide variations.
