# Supplementary material for: Canine Parvovirus in Turkey: First Whole-Genome Sequences, Strain Distribution, and Prevalence
Source: Viruses. 2023 Apr 13;15(4):957. doi: 10.3390/v15040957 (PMC10145800; doi:10.3390/v15040957)
Supplement: Supplementary file 1 [file viruses-15-00957-s001.zip › Supplement S1.pdf]

Supplement S1. Entire CPV GenBank Accession Numbers from Dogs in Turkey

No	Accession Number	Collection Year	Location	Strain	Reference
001	OQ355601	2022	Antalya (1)*	2b	This Study
002	OQ355602	2022	Antalya (2)*	2b	This Study
003	OQ355603	2022	Antalya (3)*	2b	This Study
004	OQ355604	2022	Istanbul (1)*	2b	This Study
005	OQ355605	2022	Istanbul (2)*	2b	This Study
006	OQ355606	2022	Istanbul (3)*	2c	This Study
007	OQ355607	2022	Istanbul (4)*	2b	This Study
008	OQ355608	2022	Kayseri (1)*	2a	This Study
009	OQ355609	2022	Kayseri (2)*	2b	This Study
010	OQ355610	2022	Kayseri (3)*	2b	This Study
011	OQ355611	2022	Kayseri (4)*	2b	This Study
012	OQ355618	2022	Van (1)*	2a	This Study
013	OQ355619	2022	Van (2)*	2b	This Study
014	OQ355622	2022	Yozgat (15)*	2b	This Study
015	OQ366402	2021	Ankara (2)	2b	This Study
016	OQ355596	2021	Ankara (3)*	2b	This Study
017	OQ355597	2021	Ankara (4)*	2b	This Study
018	OQ355598	2021	Ankara (5)*	2b	This Study
019	OQ355599	2021	Ankara (7)*	2a	This Study
020	OQ355600	2021	Ankara (8)*	2a	This Study
021	OQ355612	2021	Samsun (1)*	2b	This Study
022	OQ366403	2021	Samsun (3)*	2b	This Study
023	OQ355613	2021	Samsun (5)*	2a	This Study
024	OQ355614	2021	Samsun (6)*	2a	This Study
025	OQ355615	2021	Sanliurfa (1)*	2b	This Study
026	OQ355616	2021	Sanliurfa (2)*	2b	This Study
027	OQ366404	2021	Sanliurfa (3)*	2b	This Study
028	OQ355617	2021	Sanliurfa (4)*	2b	This Study
029	OQ355620	2021	Yozgat (6)*	2b	This Study
030	OQ355621	2021	Yozgat (7)*	2b	This Study
031	OM502014	2021	Burdur	2b	GenBank (Hasircioglu, 2022)
032	OM721656	2021	Kayseri	2b	Abayli et al., 2022
033	OM747857	2021	Elazig	2b	Abayli et al., 2022
034	OM747858	2021	Elazig	2b	Abayli et al., 2022
035	OM747859	2021	Elazig	2b	Abayli et al., 2022
036	OQ366405	2020	Izmir (2)*	2b	This Study
037	MW539053	2020	Izmir (1)*	2b	This Study
038	MW465343	2020	Konya	2b	GenBank (Dik et al., 2021)
039	MW465344	2020	Konya	2b	GenBank (Dik et al., 2021)
040	MW465345	2020	Konya	2b	GenBank (Dik et al., 2021)
041	MW465346	2020	Konya	2b	GenBank (Dik et al., 2021)
042	MZ197813	2020	Balikesir	2a	GenBank (Karapinar and Timurkan, 2021)

Continue…

No	Accession Number	Collection Year	Location	Strain	Reference
043	MZ197814	2020	Balikesir	2a	GenBank (Karapinar and Timurkan, 2021)
044	MZ197815	2020	Balikesir	2a	GenBank (Karapinar and Timurkan, 2021)
045	MZ197816	2020	Balikesir	2a	GenBank (Karapinar and Timurkan, 2021)
046	MZ197817	2020	Balikesir	2a	GenBank (Karapinar and Timurkan, 2021)
047	MZ197818	2020	Balikesir	2a	GenBank (Karapinar and Timurkan, 2021)
048	MZ197819	2020	Balikesir	2b	GenBank (Karapinar and Timurkan, 2021)
049	MZ197820	2020	Balikesir	2b	GenBank (Karapinar and Timurkan, 2021)
050	MZ197821	2020	Balikesir	2b	GenBank (Karapinar and Timurkan, 2021)
051	MZ197822	2020	Balikesir	2b	GenBank (Karapinar and Timurkan, 2021)
052	MZ197823	2020	Balikesir	2b	GenBank (Karapinar and Timurkan, 2021)
053	MZ197824	2020	Balikesir	2b	GenBank (Karapinar and Timurkan, 2021)
054	MZ197825	2020	Balikesir	2b	GenBank (Karapinar and Timurkan, 2021)
055	MZ197826	2020	Balikesir	2b	GenBank (Karapinar and Timurkan, 2021)
056	MZ197827	2020	Balikesir	2b	GenBank (Karapinar and Timurkan, 2021)
057	MZ391098	2020	Ankara	2a	GenBank (Kizilkoca and Tan, 2021)
058	MZ391099	2020	Ankara	2b	GenBank (Kizilkoca and Tan, 2021)
059	MZ391100	2020	Ankara	2a	GenBank (Kizilkoca and Tan, 2021)
060	MZ391101	2020	Ankara	2c	GenBank (Kizilkoca and Tan, 2021)
061	MZ545656	2020	Burdur	2b	Hasircioglu, 2023
062	MZ545657	2020	Burdur	2b	Hasircioglu, 2023
063	MZ545658	2020	Burdur	2b	Hasircioglu, 2023
064	MZ545659	2020	Burdur	2b	Hasircioglu, 2023
065	MZ545660	2020	Burdur	2b	Hasircioglu, 2023
066	MZ545661	2020	Burdur	2b	Hasircioglu, 2023
067	MZ545662	2020	Burdur	2b	Hasircioglu, 2023
068	MZ545663	2020	Burdur	2b	Hasircioglu, 2023
069	MZ545664	2020	Burdur	2b	Hasircioglu, 2023
070	MZ545665	2020	Burdur	2b	Hasircioglu, 2023
071	MZ545666	2020	Burdur	2b	Hasircioglu, 2023
072	MZ545667	2020	Burdur	2b	Hasircioglu, 2023
073	MZ545668	2020	Burdur	2b	Hasircioglu, 2023
074	MZ545669	2020	Burdur	2b	Hasircioglu, 2023
075	MZ545670	2020	Burdur	2b	Hasircioglu, 2023
076	OM747853	2020	Elazig	2b	Abayli et al., 2022
077	OM747854	2020	Elazig	2b	Abayli et al., 2022
078	OM747855	2020	Elazig	2b	Abayli et al., 2022
079	OM747856	2020	Ankara	2b	Abayli et al., 2022
080	OM721655	2019	Elazig	2b	Abayli et al., 2022
081	OM747852	2019	Elazig	2b	Abayli et al., 2022
082	MK248872	2018	Ankara	2b	Akkutay-Yoldar and Koc, 2020
083	MK248873	2018	Ankara	2b	Akkutay-Yoldar and Koc, 2020
084	MK248874	2018	Ankara	2b	Akkutay-Yoldar and Koc, 2020
085	MK248875	2018	Ankara	2b	Akkutay-Yoldar and Koc, 2020
086	MK248876	2018	Ankara	2b	Akkutay-Yoldar and Koc, 2020

Continue…

No	Accession Number	Collection Year	Location	Strain	Reference
087	MK248877	2018	Ankara	2b	Akkutay-Yoldar and Koc, 2020
088	MK248878	2018	Ankara	2b	Akkutay-Yoldar and Koc, 2020
089	MN171410	2018	Sivas	2b	Isidan and Turan, 2021
090	MN171411	2018	Sivas	2b	Isidan and Turan, 2021
091	MN171412	2018	Sivas	2b	Isidan and Turan, 2021
092	MN171413	2018	Sivas	2b	Isidan and Turan, 2021
093	MN171414	2018	Sivas	2b	Isidan and Turan, 2021
094	MN171415	2018	Sivas	2b	Isidan and Turan, 2021
095	MN171416	2018	Sivas	2b	Isidan and Turan, 2021
096	MN171417	2018	Sivas	2b	Isidan and Turan, 2021
097	MN171418	2018	Sivas	2b	Isidan and Turan, 2021
098	MN171419	2018	Sivas	2b	Isidan and Turan, 2021
099	MG780275	2017	Sanliurfa	2b	Polat et al., 2019
100	MG780276	2017	Sanliurfa	2b	Polat et al., 2019
101	MG780277	2017	Sanliurfa	2b	Polat et al., 2019
102	MG780278	2017	Sanliurfa	2b	Polat et al., 2019
103	MG780279	2017	Sanliurfa	2b	Polat et al., 2019
104	MG780280	2017	Sanliurfa	2b	Polat et al., 2019
105	MG780281	2017	Sanliurfa	2b	Polat et al., 2019
106	MG780282	2017	Sanliurfa	2c	Polat et al., 2019
107	MG780283	2017	Sanliurfa	2b	Polat et al., 2019
108	MG780284	2017	Sanliurfa	2b	Polat et al., 2019
109	MG780285	2017	Sanliurfa	2b	Polat et al., 2019
110	MG780286	2017	Sanliurfa	2b	Polat et al., 2019
111	MG780287	2017	Sanliurfa	2b	Polat et al., 2019
112	MG780288	2017	Sanliurfa	2a	Polat et al., 2019
113	MG780289	2017	Sanliurfa	2b	Polat et al., 2019
114	MG780290	2017	Sanliurfa	2b	Polat et al., 2019
115	MG780291	2017	Sanliurfa	2b	Polat et al., 2019
116	MG780292	2017	Sanliurfa	2b	Polat et al., 2019
117	MT952855	2016	Aydin	2b	GenBank (Saltik and Koc, 2020)
118	MT952856	2016	Aydin	2b	GenBank (Saltik and Koc, 2020)
119	MT952857	2016	Aydin	2b	GenBank (Saltik and Koc, 2020)
120	MT952858	2016	Aydin	2b	GenBank (Saltik and Koc, 2020)
121	MT952859	2016	Aydin	2b	GenBank (Saltik and Koc, 2020)
122	MW685563	2016	Konya	2a	Dik et al., 2022
123	MW685564	2016	Konya	2a	Dik et al., 2022
124	MW685565	2016	Konya	2a	Dik et al., 2022
125	MW685566	2016	Konya	2b	Dik et al., 2022
126	MW685567	2016	Konya	2b	Dik et al., 2022
127	MW685568	2016	Konya	2b	Dik et al., 2022
128	MW685569	2016	Konya	2a	Dik et al., 2022
129	MW685570	2016	Konya	2b	Dik et al., 2022
130	KU310544	2015	Van	2a	GenBank (Karapinar, 2015)

Continue…

No	Accession Number	Collection Year	Location	Strain	Reference
131	MG545537	2014	Van	2a	Karapinar et al., 2018
132	MG545538	2014	Van	2b	Karapinar et al., 2018
133	MG545539	2014	Van	2b	Karapinar et al., 2018
134	MG545540	2014	Van	2b	Karapinar et al., 2018
135	KX268105	2013	Ankara	2b	Gargari, 2015
136	KX268106	2013	Ankara	2b	Gargari, 2015
137	KX268107	2013	Ankara	2b	Gargari, 2015
138	KX268108	2013	Ankara	2a	Gargari, 2015
139	KX268109	2013	Ankara	2c	Gargari, 2015
140	KX268110	2013	Ankara	2a	Gargari, 2015
141	KX268111	2013	Ankara	2b	Gargari, 2015
142	KX268112	2013	Ankara	2a	Gargari, 2015
143	KX268113	2013	Ankara	2a	Gargari, 2015
144	KX268114	2013	Ankara	2a	Gargari, 2015
145	KX268115	2013	Ankara	2a	Gargari, 2015
146	KX268116	2013	Ankara	2b	Gargari, 2015
147	KX268117	2013	Ankara	2b	Gargari, 2015
148	KX268118	2013	Ankara	2b	Gargari, 2015
149	KX268119	2013	Ankara	2a	Gargari, 2015
150	KF500484	2009 - 2010	Ankara	2a	Timurkan and Oguzoglu, 2015
151	KF500485	2009 - 2010	Ankara	2a	Timurkan and Oguzoglu, 2015
152	KF500486	2009 - 2010	Ankara	2a	Timurkan and Oguzoglu, 2015
153	KF500487	2009 - 2010	Ankara	2a	Timurkan and Oguzoglu, 2015
154	KF500488	2009 - 2010	Ankara	2a	Timurkan and Oguzoglu, 2015
155	KF500489	2009 - 2010	Ankara	2a	Timurkan and Oguzoglu, 2015
156	KF500490	2009 - 2010	Ankara	2a	Timurkan and Oguzoglu, 2015
157	KF500491	2009 - 2010	Ankara	2b	Timurkan and Oguzoglu, 2015
158	KF500492	2009 - 2010	Ankara	2b	Timurkan and Oguzoglu, 2015
159	KF500493	2009 - 2010	Ankara	2a	Timurkan and Oguzoglu, 2015
160	KF500494	2009 - 2010	Ankara	2a	Timurkan and Oguzoglu, 2015
161	KF500495	2009 - 2010	Ankara	2b	Timurkan and Oguzoglu, 2015
162	KF500496	2009 - 2010	Ankara	2b	Timurkan and Oguzoglu, 2015
163	KF500497	2009 - 2010	Ankara	2a	Timurkan and Oguzoglu, 2015
164	KF500498	2009 - 2010	Ankara	2a	Timurkan and Oguzoglu, 2015
165	KF500499	2009 - 2010	Ankara	2b	Timurkan and Oguzoglu, 2015
166	KF500500	2009 - 2010	Ankara	2b	Timurkan and Oguzoglu, 2015
167	KF500501	2009 - 2010	Ankara	2a	Timurkan and Oguzoglu, 2015
168	KF500502	2009 - 2010	Ankara	2a	Timurkan and Oguzoglu, 2015
169	KF500503	2009 - 2010	Ankara	2a	Timurkan and Oguzoglu, 2015
170	KF500504	2009 - 2010	Ankara	2b	Timurkan and Oguzoglu, 2015
171	KF500505	2009 - 2010	Ankara	2b	Timurkan and Oguzoglu, 2015
172	KF500506	2009 - 2010	Ankara	2a	Timurkan and Oguzoglu, 2015
173	KF500507	2009 - 2010	Ankara	2a	Timurkan and Oguzoglu, 2015
174	KF500508	2009 - 2010	Ankara	2a	Timurkan and Oguzoglu, 2015

Continue…

* Numbers represents the isolates.
** Samples painted in gray: Partial VP2 gene presented in this study.
*** Samples painted in blue: Genome sequences presented in this study.
**** Samples painted in yellow were not fit to 426th amino acid in alignment, thus the strains written in article was accepted.
*****MK503182, MK503183, MK503184, MK503185 sequences were not in the list because of their different location in the VP2 gene region.
