# Supplementary material for: Canine Parvovirus in Turkey: First Whole-Genome Sequences, Strain Distribution, and Prevalence
Source: Viruses. 2023 Apr 13;15(4):957. doi: 10.3390/v15040957 (PMC10145800; doi:10.3390/v15040957)
Supplement: Supplementary file 1 [file viruses-15-00957-s001.zip › Supplement S2.pdf]

Supplement S2. Worldwide CPV Genomes and VP2 Gene Sequences from Dogs

No	Accession Number	Collection Year	Location	Strain	Reference
001	ON733252	2021	Hungary	2b	Miljan, 2017
002	MW653248	2021	Iran	2a	Morovvati et al., 2021
003	MW653251	2020	Iran	2b	Morovvati et al., 2021
004	MW653253	2020	Iran	2c	Morovvati et al., 2021
005	OP093952	2019	Brazil	2b	GenBank (Deus et al., 2022)
006	MZ362882	2019	Australia	2a	GenBank (Khabiri et al., 2021)
007	MZ362881	2019	Australia	2b	GenBank (Khabiri et al., 2021)
008	OM937908	2019	Egypt	2c	Ndiana et al., 2022
009	OM937913	2019	Egypt	2a	Ndiana et al., 2022
010	OM937914	2019	Egypt	2b	Ndiana et al., 2022
011	MW654969	2019	Romania	2c	Balboni et al., 2021
012	MT981021	2018	Italy	2a	Schiro et al., 2022
013	MT981023	2018	Italy	2b	Schiro et al., 2022
014	MT981024	2018	Italy	2c	Schiro et al., 2022
015	MK867450	2018	Ireland	2b	GenBank (Daly and Dunham)
016	MH545963	2018	India	2c	GenBank (Lancy et al., 2018)
017	MK895483	2018	Nigeria	2a	Ogbu et al., 2020
018	MK895490	2018	Nigeria	2c	Ogbu et al., 2020
019	MH476585	2017	China	2c	Zhuang et al., 2018
020	MH476590	2017	China	2a	Zhuang et al., 2018
021	MT165692	2017	China	2b	GenBank (Fan et al., 2020)
022	MH660909	2017	Mongolia	2c	Temuujin et al., 2019
023	OL546608	2017	Iraq	2b	Abas et al., 2022
024	OL546613	2017	Iraq	2c	Abas et al., 2022
025	OL546614	2016	Iraq	2a	Abas et al., 2022
026	MK867453	2016	UK	2b	GenBank (Daly and Dunham)
027	LC270892	2015	Japan	2b	Sehata et al., 2017
028	KU508691	2015	Australia	2c	Woolford et al., 2017
029	KP859574	2014	Croatia	2c	Miljan, 2017
030	LC214970	2013	Vietnam	2a	GenBank (Maeda and Nguyen, 2019)
031	LC214969	2013	Vietnam	2c	GenBank (Maeda and Nguyen, 2019)
032	KF539793	2012	Hungary	2a	Boros et al., 2021
033	MK867451	2012	UK	2a	GenBank (Daly and Dunham)
034	MF177286	2011	Uruguay	2c	GenBank (Grecco et al., 2018)
035	MF177280	2011	Ecuador	2b	GenBank (Grecco et al., 2018)
036	KM457102	2010	Uruguay	2a	Perez et al., 2014
037	HQ602969	2010	South Africa	2b	Dogonyaro et al., 2013
038	FJ005247	2009	Belgium	2c	Decaro et al., 2009
039	MF177227	2009	France	2c	GenBank (Grecco et al., 2018)
040	GQ865518	2008	Greece	2c	Ntafis et al., 2010
041	FJ005214	2006	Spain	2c	Decaro et al., 2009
042	FJ005260	1997	Germany	2b	Decaro et al., 2009
043	JN033694	1993	Russia	2b	GenBank (Chausov et al., 2011)

* Samples painted in gray: Whole VP2 gene.
** Samples painted in blue: Genome sequences.
