# Supplementary material for: Canine Parvovirus in Turkey: First Whole-Genome Sequences, Strain Distribution, and Prevalence
Source: Viruses. 2023 Apr 13;15(4):957. doi: 10.3390/v15040957 (PMC10145800; doi:10.3390/v15040957)
Supplement: Supplementary file 1 [file viruses-15-00957-s001.zip › Supplement S5.pdf]

Supplement S5. CPV Whole-Genome Nucleotide Variations

Nucleotide Variations																
Nucleotide Numbers	49	50	51	52	74	79	95	96	97	98	143	262	268	269	270	271
NC001539_Ref	-	-	C	C	A	-	C	-	T	C	A	C	A	A	C	C
OQ366402_Ankara2_2b	G	C	G	C	C	T	C	A	T	A	A	T	-	-	-	-
MW539053_İzmir1_2b	C	G	C	G	A	-	T	A	G	A	G	T	-	-	-	-
OQ366405_İzmir2_2b	C	G	C	G	A	-	C	-	T	C	A	T	-	-	-	-
OQ366403_Samsun3_2b	G	C	G	C	C	T	C	A	T	A	A	T	-	-	-	-
OQ366404_Sanliurfa3_2b	G	C	G	C	C	T	C	A	T	A	A	T	-	-	-	-

Nucleotide Variations																
Nucleotide Numbers	320	440	596	614	617	774	932	1004	1018	1166	1253	1553	1559	1568	1580	1604
NC001539_Ref	T	A	A	A	T	T	T	T	T	A	T	A	A	A	T	T
OQ366402_Ankara2_2b	C	G	G	G	C	T	C	C	T	A	T	A	A	A	T	T
MW539053_İzmir1_2b	T	G	A	A	T	C	T	T	T	G	T	A	A	A	C	T
OQ366405_İzmir2_2b	C	G	G	G	C	T	C	C	T	A	T	T	A	A	T	T
OQ366403_Samsun3_2b	T	G	G	G	C	T	T	T	C	A	T	A	A	A	T	T
OQ366404_Sanliurfa3_2b	C	G	G	G	C	T	C	C	T	A	C	A	G	T	T	C

Nucleotide Variations																
Nucleotide Numbers	1652	1655	1754	1811	1875	1893	1904	1949	1955	2002	2011	2020	2022	2027	2058	2065
NC001539_Ref	T	T	G	G	T	G	T	A	A	C	G	T	G	A	C	T
OQ366402_Ankara2_2b	T	C	G	G	T	G	T	A	C	C	G	C	A	G	C	T
MW539053_İzmir1_2b	C	T	G	A	T	G	C	G	A	C	G	T	G	G	C	C
OQ366405_İzmir2_2b	T	C	G	G	T	A	T	A	C	C	G	C	A	A	C	T
OQ366403_Samsun3_2b	T	T	G	G	C	G	T	A	C	T	G	T	G	A	G	T
OQ366404_Sanliurfa3_2b	C	T	A	G	T	G	T	A	C	C	A	C	A	G	C	T

Continue…

Nucleotide Variations																
Nucleotide Numbers	2198	2240	2292	2576	2707	2765	2909	2920	2936	2947	2991	3048	3068	3091	3092	3173
NC001539_Ref	G	T	G	G	A	A	T	C	G	T	C	A	C	T	T	A
OQ366402_Ankara2_2b	A	C	C	G	A	A	T	C	A	T	C	T	C	C	T	A
MW539053_İzmir1_2b	A	T	G	G	G	A	T	C	A	T	C	T	C	C	C	A
OQ366405_İzmir2_2b	A	C	C	A	A	C	T	C	A	T	C	T	C	C	T	A
OQ366403_Samsun3_2b	A	C	C	G	A	A	G	G	A	G	T	T	T	C	T	A
OQ366404_Sanliurfa3_2b	A	C	C	G	G	A	T	C	A	T	C	T	C	C	T	G
Nucleotide Variations																
Nucleotide Numbers	3194	3215	3323	3377	3386	3521	3545	3583	3589	3678	3688	3702	3737	3759	3760	3764
NC001539_Ref	G	A	T	T	A	T	A	C	T	T	C	G	A	T	A	T
OQ366402_Ankara2_2b	G	A	C	C	A	C	G	C	A	G	G	T	G	A	T	T
MW539053_İzmir1_2b	G	A	C	T	A	C	G	C	A	G	G	T	G	A	T	T
OQ366405_İzmir2_2b	G	A	C	T	A	C	G	C	A	G	G	T	G	A	T	T
OQ366403_Samsun3_2b	A	A	C	T	C	T	A	C	T	G	G	T	G	A	T	T
OQ366404_Sanliurfa3_2b	G	G	C	T	A	C	G	T	A	G	G	T	G	G	T	C
Nucleotide Variations																
Nucleotide Numbers	3888	4064	4065	4107	4124	4145	4262	4448	4511	4557	4577	4593	4607	4619	4624	4627
NC001539_Ref	T	G	A	A	T	T	A	T	A	G	A	A	T	G	T	T
OQ366402_Ankara2_2b	G	A	G	G	C	T	A	C	A	-	C	-	C	-	T	T
MW539053_İzmir1_2b	G	A	G	G	C	T	A	C	A	G	A	A	T	G	G	T
OQ366405_İzmir2_2b	G	A	G	G	C	T	G	C	A	G	A	A	T	G	G	T
OQ366403_Samsun3_2b	G	A	G	G	C	A	A	C	A	-	A	A	C	T	-	A
OQ366404_Sanliurfa3_2b	G	A	G	G	C	T	A	C	G	-	C	-	C	-	T	T

Continue…

Nucleotide Variations																
Nucleotide Numbers	4629	4633	4660	4687	4706	4715	4768	4776	4777	4793	4795	4807	4808	4810	4811	4812
NC001539_Ref	T	T	-	T	-	G	G	A	G	T	T	G	A	C	-	-
OQ366402_Ankara2_2b	T	T	T	C	G	T	G	A	G	A	C	A	T	T	T	G
MW539053_İzmir1_2b	T	T	T	C	G	T	-	A	G	T	T	G	A	C	-	-
OQ366405_İzmir2_2b	T	T	T	C	G	T	-	A	G	T	T	G	A	C	-	-
OQ366403_Samsun3_2b	G	A	T	C	G	T	G	A	G	T	T	A	T	T	T	G
OQ366404_Sanliurfa3_2b	T	T	T	C	G	T	G	T	T	T	T	A	T	T	T	G

Nucleotide Variations																
Nucleotide Numbers	4813	4814	4815	4816	4817	4818	4819	4820	4821	4824	4826	4829	4830	4831	4833	4838
NC001539_Ref	-	-	-	-	-	-	-	-	A	T	G	C	A	T	G	T
OQ366402_Ankara2_2b	T	A	C	T	T	G	T	A	C	G	T	A	G	G	T	A
MW539053_İzmir1_2b	-	-	-	-	-	-	-	-	A	T	G	C	A	T	G	T
OQ366405_İzmir2_2b	-	-	-	-	-	-	-	-	A	T	G	C	A	T	G	T
OQ366403_Samsun3_2b	T	A	C	T	T	G	T	A	T	G	T	A	G	G	T	A
OQ366404_Sanliurfa3_2b	T	A	C	T	T	G	T	A	C	G	T	A	G	G	T	A

Nucleotide Variations													
Nucleotide Numbers	4885	4904	4914	4916	4919	4968	5077	5080	5081	5082	5088	5234	5297
NC001539_Ref	T	T	A	A	T	T	G	G	C	-	A	T	A
OQ366402_Ankara2_2b	T	T	A	A	T	T	T	A	A	G	A	C	A
MW539053_İzmir1_2b	A	G	A	A	T	T	G	G	C	-	A	C	A
OQ366405_İzmir2_2b	A	G	A	A	T	T	G	G	C	-	A	C	A
OQ366403_Samsun3_2b	T	T	A	A	C	T	T	A	A	G	A	C	A
OQ366404_Sanliurfa3_2b	T	T	T	T	T	A	T	A	A	G	G	C	G

* The gray-shaded nucleotides are different to the reference
