# Supplementary figures and images for: Canine Parvovirus in Turkey: First Whole-Genome Sequences, Strain Distribution, and Prevalence
Source: Viruses. 2023 Apr 13;15(4):957. doi: 10.3390/v15040957 (PMC10145800; doi:10.3390/v15040957)

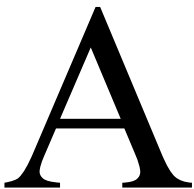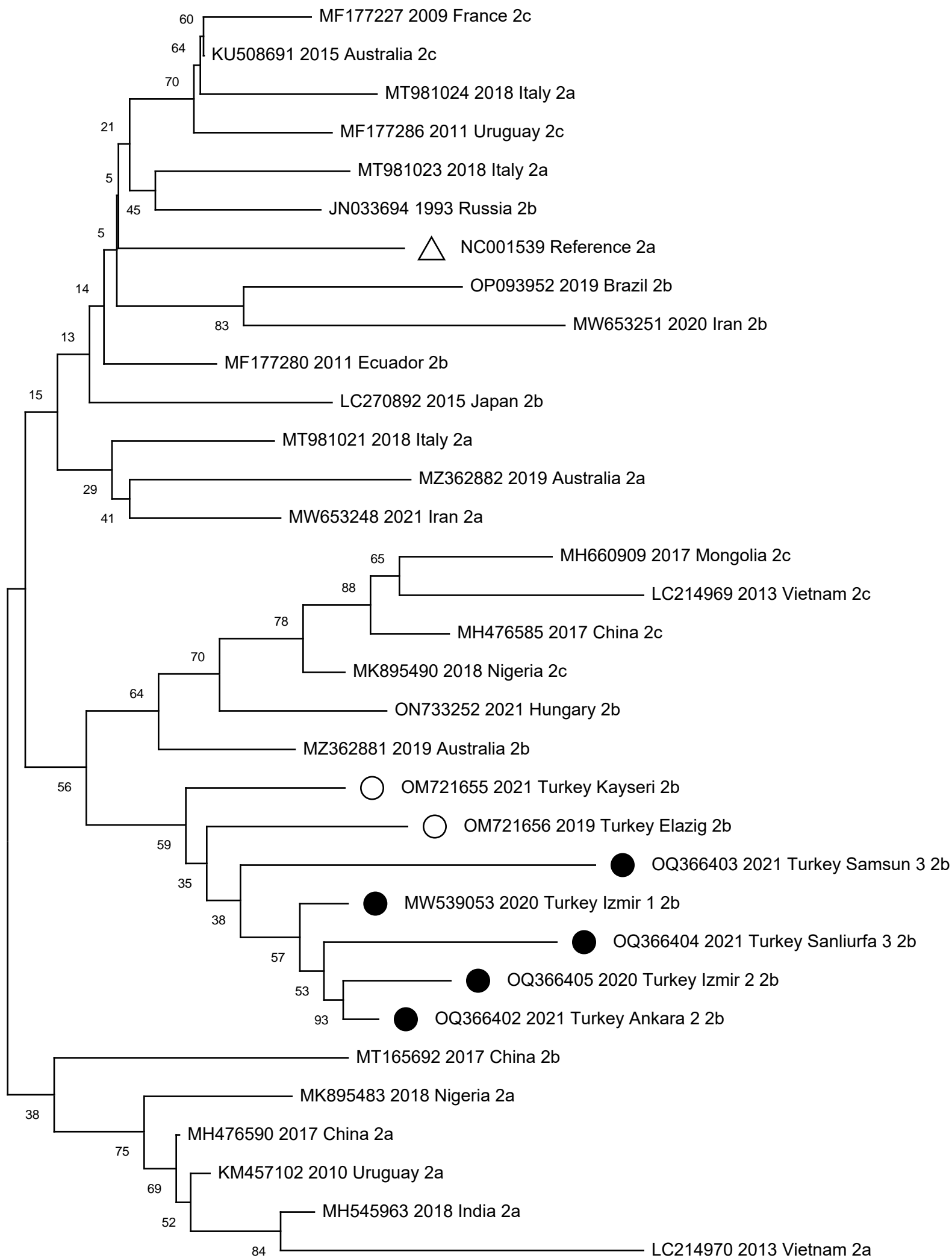

0.0020

B

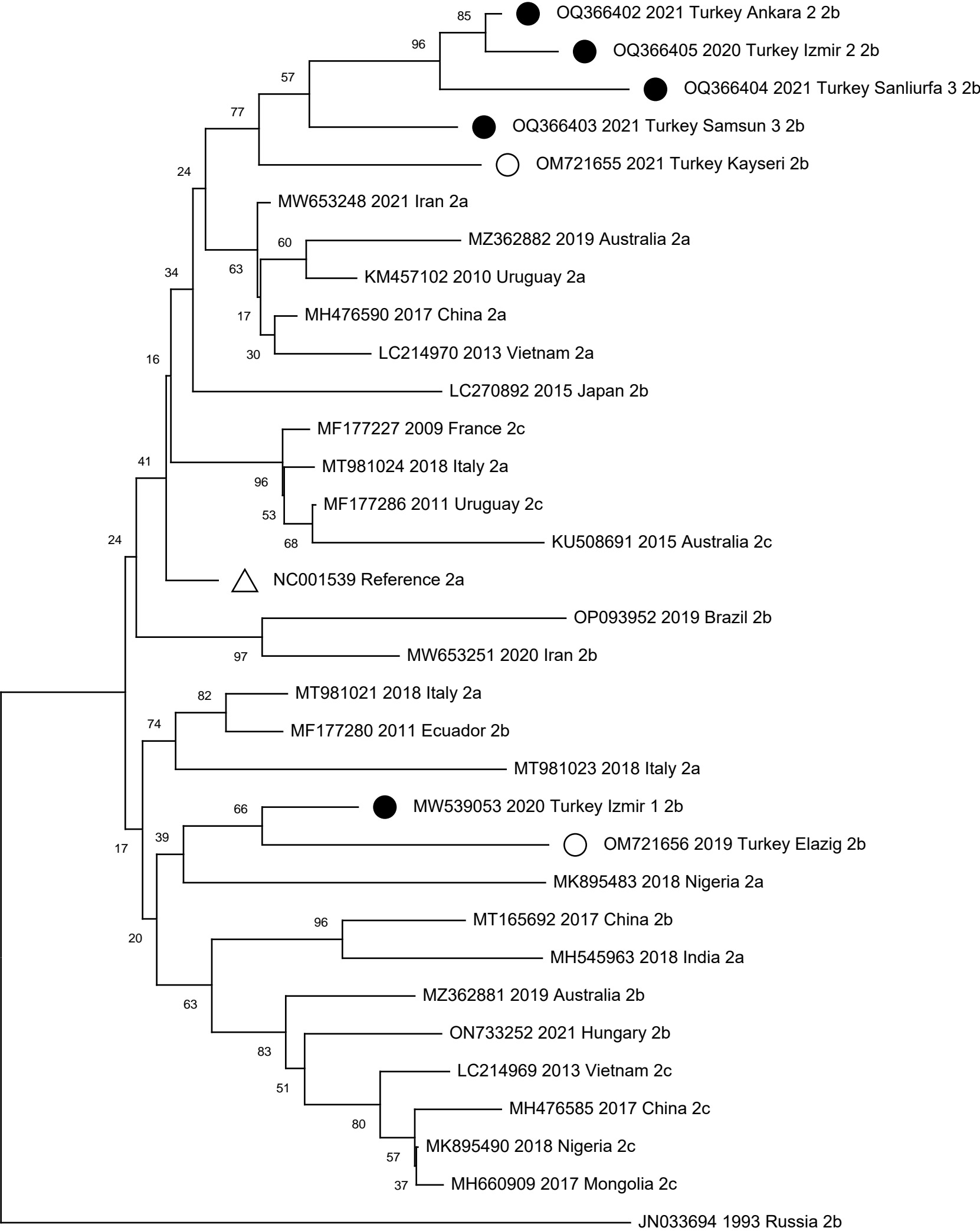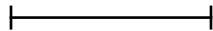

0.0020

Supplement: Supplementary file 1 [file viruses-15-00957-s001.zip › Supplement S3.pdf]

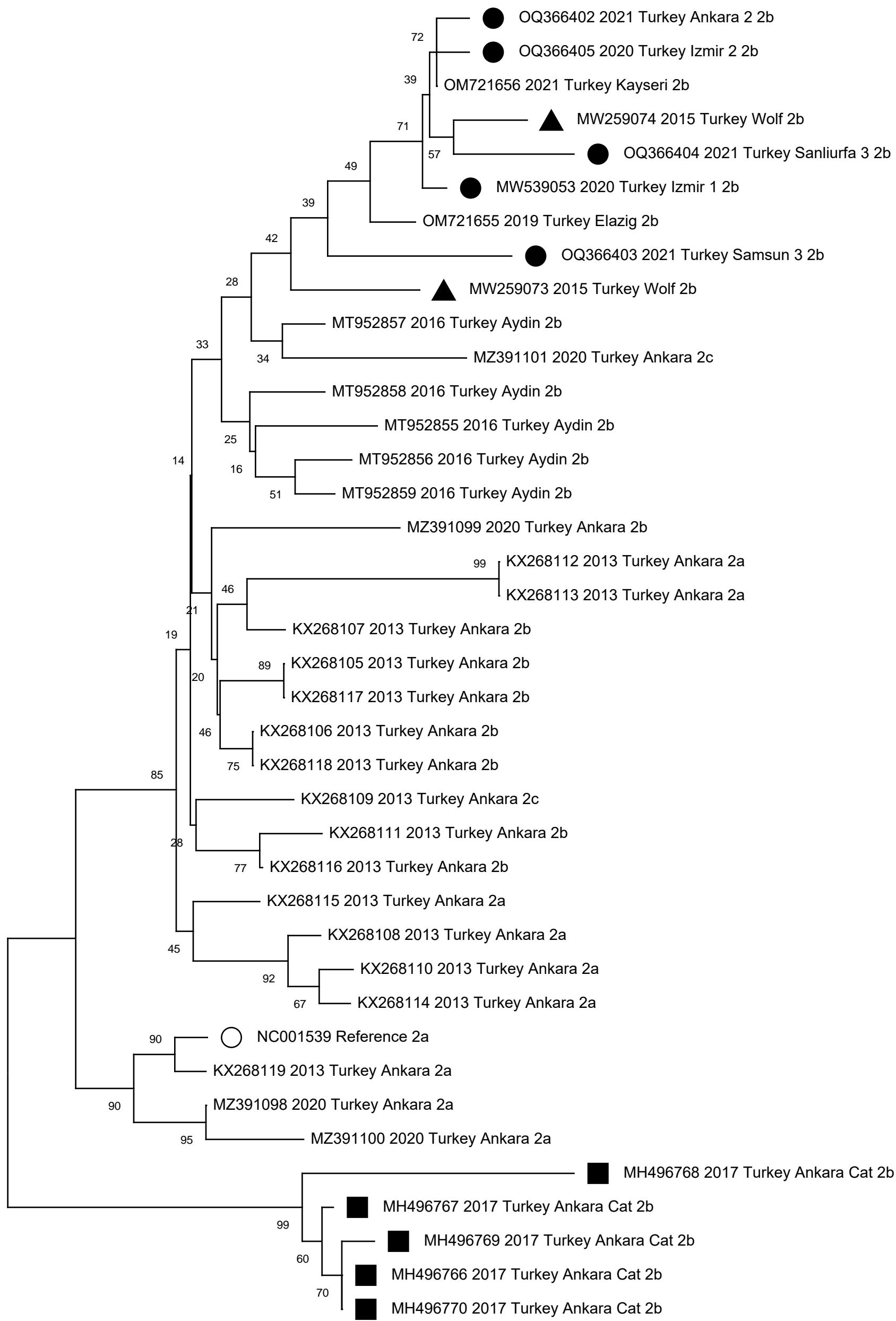

Supplement: Supplementary file 1 [file viruses-15-00957-s001.zip › Supplement S4.pdf]
